# A Review of Patient Preferences for Osteoporosis Drug Treatment

**DOI:** 10.1007/s11926-015-0533-0

**Published:** 2015-08-19

**Authors:** Mickaël Hiligsmann, Sandrine P. G. Bours, Annelies Boonen

**Affiliations:** Department of Health Services Research, School for Public Health and Primary Care (CAPHRI), Maastricht University, P.O. Box 616, 6200 MD Maastricht, The Netherlands; Department of Internal Medicine, CAPHRI, Maastricht University Medical Centre, Maastricht, The Netherlands

**Keywords:** Crossover designs, Discrete-choice experiment, Drug therapy, Osteoporosis, Preferences, Review, Satisfaction

## Abstract

Poor medication adherence is a major problem in chronic diseases such as osteoporosis that may partially be due to unaddressed patient values and preferences. Data on patient preferences could help clinicians to improve medication adherence and could also be useful in policy decisions and guideline development. This paper aims to identify literature reporting on the preferences of patients for osteoporosis drug medications. Several methods have been used to elicit patient preferences for medications and their characteristics including qualitative research, survey with ranking/rating exercises, discrete-choice experiments and clinical studies (crossover designs, open-label study). All these studies revealed that osteoporotic patients have preferences for medications and their attributes, in particular for less-frequent dosing regimens. Interestingly, variations in the preferences of patients were observed in most studies, suggesting the importance to take into account individual preference in decision-making to improve osteoporosis care.

## Introduction

Osteoporosis represents an increasing public health problem, especially in the Western world. For the year 2010, it was estimated that about 27.5 million of people from the European Union have osteoporosis [1], resulting in approximately 3.5 million new fractures. Despite the fact that several drugs have demonstrated to be safe and effective in reducing the risk of fractures [2], adherence to medications remains poor and suboptimal [3], with substantial clinical and economic implications [4]. Poor adherence to therapy may partially be due to unaddressed patient values and preferences [5].

Understanding what patients prefer and involving them in clinical decision-making could lead to improved satisfaction with therapy and hence medication adherence [6]. Patient perceptions and preferences with osteoporosis medications were shown to impact adherence behaviour [7••] and discontinuation rates [8]. The patient’s perspective is now becoming increasingly important in the design and assessment of healthcare interventions [9]. Patients want to be informed by their doctors and play an active role in clinical decision-making [10]. Therefore, in recent years, there has been a growing interest in studies to elicit preferences for healthcare interventions. There are different ways to elicit patient preferences including qualitative research, survey with ranking/rating exercises, discrete-choice experiments and clinical studies (such as crossover or open-label study).

With the development of new osteoporosis medications that differ mainly according to mode of administration, it is not surprising that several studies have been conducted to elicit patient preferences for osteoporosis medications and understand the so-called attributes or factors that contribute to preferences. Reviewing these studies and reporting their findings could have substantial potential for clinicians towards improving poor medication adherence and could also be very useful for policy decisions and guideline development [11]. This paper was therefore designed to review studies that assessed preferences in osteoporosis. A secondary aim was to discuss how preference could be incorporated in clinical decision-making.

## Patients’ Preferences for Osteoporosis Medications

A review of published studies that describe patient preferences for osteoporosis drug treatment was conducted in PubMed. The search terms were ‘preference* [title/abstract] OR satisfaction [title/abstract] AND ‘osteoporosis [MESH term]’, and all articles published until May 1, 2015, were included. We only included original research that looks at preferences for osteoporosis medications and/or medication attributes. We followed the operational definition of preference given by Joy et al. [11]: ‘given a choice, the selection of an alternative’. We therefore included studies that looked at the choice of patient between alternatives. We also included preference studies that assessed preferences for medication attributes using hypothetical treatment options and studies that were interested in preferences for medication characteristics. Non-medication interventions were excluded. Abstract and title screening was initially performed, followed by a full-text screening. References of identified articles were searched for additional articles and completed by authors’ knowledge of the published literature. Data were collected on study authors, country, publication year, method, sample size, funding and main findings. The number of medication attributes was included for surveys, and interventions were included for clinical studies.

The PubMed search yielded 246 articles. A total of 23 articles met our inclusion criteria. Ten studies used a questionnaire or interviews to elicit preferences for medication attributes. Among these, 4 studies specifically aimed to identify the attributes that play a role to preferences of osteoporotic patients for medication attributes, 4 used conjoint analysis methods to elicit the trade-offs patients make between attributes and 2 were interview-based surveys to elicit preferences between two medications. The remaining 13 articles investigated patient preferences between two real-life interventions using an open-label study (*n* = 7) or crossover design (*n* = 6). Tables 1 and 2 summarize the characteristics and main findings of all these studies.

**Table 1:** Study characteristics and findings of stated-preference studies eliciting patients’ preferences for osteoporosis medications

First author	Country	Publication year	Method	Sample size	Funding	Number of attributes (or medications)	Main findings
Importance of medication attributes							
Weiss [12]	USA	2006	Survey (ranking and rating)	3368	Merck & Co.	8	Order of importance: effectiveness, side effects, interactions, out-of-pockets costs, time on market, dosing frequency, formulation, dosing frequency
Duarte [13]	France, Germany, Mexico, Spain and UK	2007	Survey (ranking and rating)	3000	NR	7	Order of importance: effectiveness, side effects, out-of-pocket costs, dosing frequency, formulation, time on market, dosing procedure
Hiligsmann [14•]	Belgium and Netherlands	2013	Nominal group technique	26	Amgen	12	Order of importance: effectiveness, side effects, frequency of administration and mode of administration, out-of-pocket cost, sequential therapy, place of administration, time on market, branded/generic, mono or combination, mode of action, cost for society
Silverman [15•]	USA	2013	MaxDiff ranking	367	Novartis Pharmaceuticals	4	Order of importance: efficacy, safety, cost and convenience
Conjoint analyses							
Fraenkel [18]	USA	2006	ACA	212	NR	4	Patients’ treatment preferences were strongly influenced by route of administration. Patients’ preferred treatment option, across all simulations, was bisphosphonates
de Bekker-Grob [17]	Netherlands	2008	DCE	120	Public	5	All attributes (effectiveness, side effects (nausea), total treatment duration, route of drug administration, and out-of-pocket costs) were important Patients preferred a tablet once a month than tablet once a week or injection once a month or every 4 months
Darba [16]	Spain	2011	DCE	166	NR	3	All attributes (route of administration, place of administration and costs) were important Patients preferred subcutaneous injection one per day rather than intravenous injection once per year
Hiligsmann [19••]	Belgium	2014	DCE	257	Amgen	5	All attributes (effectiveness, side effects, mode and frequency of administration and costs) were important Patients preferred either an oral monthly tablet or 6-month subcutaneous injection above weekly oral tablets, 3-month subcutaneous, 3-month intravenous or yearly intravenous injections Patients disliked being at risk of gastrointestinal disorders more than being at risk of skin reactions and flu-like symptoms
Structured interviews							
Gold [23]	USA	2006	617 women currently using bisphosphonates	Interviews	Alliance for Better Bone Health	Two medications: weekly vs. monthly bisphosphonate therapy	More patients preferred weekly therapy over monthly therapy (82 vs. 18 %), after receiving information about fracture efficacy differences
Keen [22]	UK, Germany, France, Spain and Italy	2006	1253 currently taking a weekly bisphosphonate or had no current or prior history of bisphosphonate therapy	Interviews	NR	Two medications: weekly vs. monthly bisphosphonate therapy	82 % preferred weekly bisphosphonate and 18 % monthly therapy Efficacy was the most commonly cited reason for this preference Overall, 69 % of patients intended to ‘definitely/probably’ use the weekly therapy compared with 34 % of women who rated the monthly therapy similarly

*ACA* adaptive conjoint analysis, *DCE* discrete choice experiment

**Table 2:** Study characteristics and findings of clinical studies eliciting patients’ preferences for osteoporosis medications

First author and publication year	Country	Study duration	Sample size	Funding	Interventions	Outcomes
Crossover designs						
Freemantle [25], 2012	USA and Canada	2 years	250 (221 in the second year)	Amgen	Denosumab vs. alendronate	Non-adherence less for denosumab (first year 11.9 vs. 23.4 %; second year 7.5 vs. 36.5 %) BMQ scores higher for denosumab Preference denosumab over oral therapy in 92.4 %
Chung [29], 2009	South Korea	6 months	365	GSK Korea	Monthly oral ibandronate vs. weekly risedronate	Preference for once-monthly ibandronate in 74.8 % of patients Once-monthly ibandronate more convenient (84.2 %) than weekly regimen (15.8 %)
Emkey [27], 2005	USA	6 months	342	Hoffmann-LaRoche, Ltd. GlaxoSmithKline	Weekly alendronate vs. monthly oral ibandronate	Preference for once-monthly ibandronate in 71.4 % Once-monthly ibandronate more convenient (74.6 %)
Hadji [30], 2008	USA, France and Germany	6 months	350 (321 in mITT)	Hoffmann-LaRoche Ltd. GlaxoSmithKline	Weekly alendronate vs. monthly oral ibandronate	Preference for once-monthly ibandronate in 70.6 % Once-monthly ibandronate more convenient (76.6 %)
Kendler [34], 2004	19 countries in Europe, Middle East, the Americas and Asia-Pacific	8 weeks	406	Merck	Daily vs. weekly alendronate	Preference for once-weekly dosing regimen in 84 % Once-weekly regimen more convenient (87 %) 84 % would be more willing to take it for a long period of time Adherence, 97 % for weekly tablets and 86 % for daily tablets
Simon [31], 2002	USA	8 weeks	288	Merck & Co., Inc.	Daily vs. weekly alendronate	Preference for once-weekly regimen in 86.4 %, once-daily regimen in 9.2 %, no preference in 4.4 % Once-weekly regimen more convenient in 89.0 %, once-daily regimen in 7.7 % Long-term compliance believed to be better for once-weekly regimen in 87.5 % and once-daily regimen in 8.5 %
den Uyl [24], 2010	The Netherlands	28 days	102	Nycomed Group AS/Roskilde, Denmark	Chewable tablet vs. sachet calcium and vitamin D	The mean number of days on drug was 12.8 days for the chewable tablet and 13.5 days for the sachet Preference for chewable tablet 67 %, for sachet 19 %, no preference (15 %) Acceptability variables (removing the dose from container, taking the dose, taste, time spent taking the dose and general convenience of taking the dose) were higher for the chewable tablet
Open-label study						
Kendler [28], 2011	USA and Canada	1 year (first year of the study of Freemantle)	250	Amgen	Denosumab vs. alendronate	Preference lower for alendronate, both at baseline and after 6 months Satisfaction higher for denosumab and treatment bother higher for alendronate Non-adherence: 23.4 % for alendronate and 12.7 % for denosumab Risk ratios for denosumab compared with alendronate at 12 months 0.58 for non-adherence, 0.48 for noncompliance, 0.54 for non-persistence
Bonnick [26], 2009	USA	6 months	1678	Roche and GlaxoSmithKline	Switch from weekly oral alendronate or risedronate to monthly oral ibandronate	Satisfaction scores improved 9 points by month 6 despite the high mean baseline summary scores (80.1 points) Convenience, overall satisfaction and quality of life domain scores improved. Increased satisfaction reported by 70.4 % of patients at month 6 Preference of once-monthly ibandronate 73.6 % (87.0 % of 1460 who completed Pref-Q) after 6 months
Kastelan [35], 2008	Croatia	6 months	258	Not reported	Switch from weekly oral alendronate or risedronate to monthly oral ibandronate	Satisfaction higher with the monthly than weekly dosing Preference for once-monthly dosing in 94.7 %, once weekly regimen in 2.0 % and no preference in 3.3 % Quality of life improved in 85.9 % of patients, did not change in 9.3 %, worsened in 1.2 % with once-monthly ibandronate Once-monthly regimen more convenient in 93.0 % and once-weekly in 4.1 %
Vlak [32], 2011	Croatia	6 months	385	Not reported	Switch from weekly oral alendronate or risedronate to monthly oral ibandronate	Increase in values of 4 domains of OPSAT-Q and CSS after switch
Palacios [33], 2015	Europe, USA, Australia and Canada	1 year	1703	Amgen	Denosumab vs. monthly oral ibandronate (TTI) or vs. weekly oral risedronate (TTR) Patients previously treated with daily or weekly oral (TTR) or any (TTI) bisphosphonates with suboptimal adherence	Compared with baseline, patients in both treatment groups reported greater satisfaction in all TSQM domains at 6 and 12 months and changes from baseline persisted from month 6 to month 12 Changes in TSQM scores for all four domains were significantly greater in patients who transitioned to denosumab at all post-baseline time points, especially in the domains of convenience and global satisfaction
McClung [36], 2007	USA	1 year	225	Novartis Pharma AG, Basel	Weekly oral alendronate vs. zoledronic acid intravenous infusion once-yearly	Preference for once-a-year infusion by 78.7 %; for once-a week oral regimen by 9 %; no preference in 11.8 %

### Importance of Medication Attributes

Four studies assessed the importance of osteoporosis medication attributes [12, 13, 14•, 15•]. The PREFER study was a large-scale survey that was conducted in the USA [12] and in five other countries [13]. Participants from these countries were administered an online questionnaire in which they were asked to rate and rank the importance of seven (or eight) medication attributes in determining their preferences for osteoporosis medications. In Hiligsmann et al. [14•], patient group discussions were conducted to prioritize a list of 12 potentially important medication attributes identified from the literature and discussion with clinicians. A total of five focus groups were conducted in the Netherlands and Belgium. Silverman et al. [15•] asked participants to evaluate the relative weight of specific statements using MaxDiff analyses. In MaxDiff analyses, participants receive a list of statements and are asked to indicate the most (and the least) important one.

As shown in Table 1, in all these studies, drug effectiveness was the most important attribute for the patients followed by side effects. Out-of-pocket costs and mode/frequency of administration were also considered important. Variations in the preferences of patients were reported. Hiligsmann et al. [14•] revealed different findings across the focus groups. The country system could also influence the preferences. In the Netherlands, where patients have no out-of-pocket contribution, the cost attribute was not as important as in Belgium [14•]. Silverman et al. [15•] also reported a significant impact of age, income, education and prior fractures on overall ranking but not of racial/ethnic differences.

### Conjoint Analyses Studies

Four studies used conjoint analysis methods to assess trade-off that patients make between medication attributes [16–18, 19••]. Fraenkel et al. [18] used ‘adaptive conjoint analyses’ to determine the treatment preferences for oral bisphosphonates by frequency of administration. Results suggested that preferences are strongly influenced by route of administration. Three discrete-choice experiments (DCEs) were conducted [16, 17, 19••]. A DCE is a form of conjoint analysis that describes an intervention by its attributes (e.g. effectiveness, side effects, costs) and reports how patient’s preference for an intervention is influenced by the type and levels of these attributes [20]. In the DCEs, patients were asked to choose between two unlabelled drug treatments (A and B) and sometimes a third ‘no treatment’ option. De Bekker-Grob et al. [17] evaluated the preferences of patients for osteoporosis treatments in the Netherlands. All attributes (i.e. effectiveness, side effects, treatment duration, route of drug administration, and out-of-pocket costs) were shown to be important. The timing of administration was however limited to a maximum of 4 months. New therapies with longer dosing intervals have recently become available, and preferences for these new administration schemes should be investigated. Darba et al. (2010) also used a DCE to investigate the importance of different treatment aspects in Spain. Only three attributes were however included in the experimental design (i.e. type of drug administration, place of administration, cost) and were all important. More recently, Hiligsmann et al. [19••] conducted a DCE with Belgian patients. This study confirms expected results that patients prefer treatments offering higher efficacy, lower costs and less-frequent dosing regimens. This study does, in addition, evaluate additional administration routes and the side effects deemed important by patients [21•]. Patients had a preference for a 6-month subcutaneous injection and weekly oral tablet compared with oral tablet and yearly intravenous. Using a mixed logit model that allows coefficients to vary between patients, this study also revealed that preferences could substantially differ between patients.

### Structured Interviews

There were 2 studies conducting structured interviews by phone [22] or from a panel of patients currently using oral bisphosphonates [23]. In Keen et al. [22], 50 % of the participants of the interviews previously or currently used oral bisphosphonates. Participants were asked to choose between two oral treatments, a weekly oral tablet with proven efficacy at the spine and hip and a monthly oral tablet with no proven efficacy at the hip. Most participants chose the treatment with proven efficacy at both hip and spine, even though the other choice was the less frequently dosed treatment [22, 23]. Previous bisphosphonate use did not influence preference; however, it affected ‘intention to use’ significantly in a positive direction [22].

### Clinical Studies

Most preference studies identified in this review were clinical studies where patients were asked to describe their preferences and satisfaction to treatment that they had received or tested. Most studies have focused on the influence of dosing frequency (daily, weekly, monthly, bi-annually or annually) for therapy on patient preference. Studies were either crossover or open-label studies.

In the clinical studies, the following questionnaires were used: Beliefs about Medicines Questionnaire (BMQ, including 22 questions in the following major domains: the necessity of the prescribed medication to manage osteoporosis now and in the future, concerns about the potential adverse effects of taking the prescribed medication, preference for one medication over the other) [24, 25], Preference Satisfaction Questionnaire (PSQ, measuring preference, pill satisfaction, injection satisfaction, pill bother and injection bother) [25–28], questionnaires for preference adapted from Balto I and II [29–31], Osteoporosis Patient Satisfaction Questionnaire (OPSATQ) [26, 32] and Treatment Satisfaction Questionnaire for Medication (TSQM; consisting of 14 items to assess an individual’s perception of effectiveness, side effects, convenience and global satisfaction) [33].

Overall, the crossover studies showed a preference for 6-monthly injections or once-monthly oral bisphosphonates over once-weekly oral bisphosphonates [25, 27, 29, 30], or a preference for once-weekly oral bisphosphonates over daily oral bisphosphonates [31, 34]. One study compared chewable and sachet calcium/vitamin D supplementation and found a preference for the chewable variant [24]. The sequence of treatments did not influence preferences [27, 30, 31]. Participants considered the lower dosing frequency of oral bisphosphonates to be more convenient [27, 29, 30, 34]. Participants were asked for perceived long-term adherence in 3 studies [25, 31, 34] and thought that the less-frequent dosing regimen would lead to better long-term adherence. Some of the crossover studies also tested for adherence [24, 25, 34] and found a lower adherence in the less preferred treatment.

The open-label studies included patients who had been on weekly bisphosphonates and who were switched to a monthly regimen [26, 32, 35] or to another bisphosphonate or denosumab [33] and showed a preference for the less frequently administered treatment. Kendler et al. (2011) found no difference in perceived necessity of osteoporosis treatment between 6-monthly injections or weekly oral bisphosphonates at baseline, but after 6 months, the perceived necessity was higher in the denosumab group. Concern about side effects did not differ between groups. In the study of Kastelan et al. [35], compared to patients who refused to participate in the study (*n* = 67) and those who did not start monthly ibandronate according to the suggestion of their attending doctor (*n* = 15), enrolled patients (*n* = 258) stated less satisfaction with the weekly bisphosphonates at baseline (*p* < 0.001) and were more likely to have adverse events during weekly treatment. Patients reported that the once-monthly dosing regimen better fitted their lifestyle and they preferred the less-frequent dosing and thought it to be an easier regimen to follow at long term. One study was a randomized clinical trial comparing a weekly oral and a yearly intravenous bisphosphonate, and patients completed the questionnaire without knowing their randomization. The preference for the yearly administered medication was highest [36].

## Use of Preferences in Clinical Decision-Making

Over the last century, the provision and evaluation of medical care saw several paradigm shifts. While medicine relied for ages on experience-based medicine, a shift towards evidence-based medicine followed by evidence-based practice was seen in the twentieth century. While evidence-based medicine and practice was largely based on efficacy/effectiveness, safety and increasingly cost-effectiveness, Carr et al. [37] already recognized in 2001 that diseases do not exist in a medical vacuum and that it is impossible to separate disease from an individual’s personal and social context. On this line, the twenty-first century is becoming the age of preference-based medicine in which the clinicians become the experts on the medical options, while patients (and their families) bring in their values and preferences. This ‘patient centered care’ required a whole new area of research into patient-reported outcomes and even more into methods to assess preferences and in approaches to incorporate preferences in the medical office. Preference studies on medication choices, as reviewed above, are valuable to assess which treatments or which treatment attributes are valued (or not) by patients at the group level. Such studies are of great value as they learn that patients’ values differ from those of doctors and reveal what aspects of health, treatment or care patients specifically value. Such insights can help when developing treatment guideline (that largely ignores preferences) but can also be an impetus to account for patient needs when developing drugs, also for seemingly futile aspects like treatment administration and frequency of dosing. However, the study by Hiligsmann also emphasized that preferences elicited at the group level show large variance around the estimated coefficients, indicating heterogeneity in preferences between patients. Therefore, in clinical practice, tools are needed that can reveal preferences of individual patients in decisions and support shared decision-making. In osteoporosis, several decision aids are available already, some supporting the patients in the decision on whether or not to start a treatment for (prevention of) osteoporosis, some supporting the decision to choose a specific drug, characterized by its different attributes, and others both aspects of decision [5]. Such decision tools can be either applied in the consultation room by the physician and research nurse or can be used by patients themselves as self-standing online tools. Although a systematic Cochrane review on decision aids showed evidence that decision aids improve knowledge, reduce decisional conflict and succeed in aligning received care with personal values [38], another study revealed that in 1000 office visits in which 3500 medical decisions were made, less than 10 % of decisions met the minimum for informed decision-making [39]. Thus, it appears that even when we really believe in preference-based care, lots of work is still needed [40].

## Discussion

Our review identified several studies that assessed preferences of osteoporotic patients for medications. Different methods have been used including clinical studies and stated-preference methods. In clinical studies, different questionnaires are available to test for preferences and/or convenience. Eliciting preferences seems however not always a straightforward process, as revealed by studies comparing preferences for weekly and monthly oral tablet. Crossover or open-label studies comparing once-monthly and once-weekly oral bisphosphonates revealed a preference for the once-monthly oral tablet [27, 29, 30, 35] in patients who had taken both regimens. However, when patients were informed about the limited evidence of efficacy of the once-monthly oral regimen, the preference was in opposite direction with most patients preferring once-weekly oral bisphosphonate with proven efficacy at both the spine and the hip [22, 23]. In addition, in recent years, an increased use of stated-preference methods like DCEs to elicit preferences in healthcare has been seen [41], which could be interesting to reveal preferences even before receiving a therapy. Formal stated-preference methods use survey/question to elicit patient preferences for hypothetical treatment options in an experimental framework and allow to assess the importance of medication attributes and trade-offs that patients make between them. Alternatively, revealed preference methods are based on observed data related to patients’ actual choice. Patients’ choice in healthcare however does not often reflect what the patient prefers given imperfect information and revealed preference methods can also not reveal which factors influence the decisions.

One limitation of the design of the clinical studies (especially in open-label studies) is the selection bias. Some of the open-label studies that switched from a baseline regimen to the study medication included patients with higher rates of dissatisfaction and more side effects at baseline, whereas the patients who were not included did better on the baseline regimen [34]. There is no information available about the preference of the patients who were not included, who did have less side effects and who might have a different preference. Anyhow, in the crossover and open-label studies, the preferred regimen was associated with higher convenience and higher observed adherence. Moreover, patients perceived that their long-term adherence would be better with the preferred regimen. Taking into account the preference of the individual patient would implicate explaining about the different dosing regimens and means of application, the potential benefits of the different regimen and the potential side effects. Prescribing the preferred treatment could improve (long-term) adherence; however, we are lacking evidence of clinical studies.

There are some potential limitations to our review. First, although we reviewed the literature using PubMed, we did not perform a complete systematic review following the PRISMA statement. Since our key words were perhaps limited and we limited our search to one database, we probably missed some papers that however would not have impacted our main findings. Second, we did not provide a quality assessment of the papers. Currently, there are no general guidelines for assessing the quality of stated-preference studies [14•]. Despite these potential limitations, reviewing preferences provide interesting information for clinicians and decision-makers which should be aware that osteoporotic patients could reveal preferences for medications and their characteristics. Furthermore, taking into account these preferences when choosing for a treatment for osteoporosis could lead to a higher adherence and compliance. With the increasing importance of the patient perspective and as more studies report on patient preferences, we could also expect that reviews of preferences will be more often made in the future.

## Conclusions

This review revealed that osteoporotic patients report preferences for medications and their characteristics. Although effectiveness and side effects were, not surprisingly, important considerations in treatment preferences, frequency of administration (and in particular less-frequent dosing regimens) is also highly valued by patients, as suggested by Bansback et al. in rheumatic diseases [42•]. Understanding patients’ preferences and incorporating them in clinical decision-making could lead to improved osteoporosis care. Patient perceptions and preferences with osteoporosis medications have been shown to impact adherence behaviour [7••] and discontinuation rates [8].
